# Limited Adaptation of *Staphylococcus aureus* during Transition from Colonization to Invasive Infection

**DOI:** 10.1128/spectrum.02590-21

**Published:** 2023-06-21

**Authors:** Anna K. Räz, Federica Andreoni, Mathilde Boumasmoud, Judith Bergada-Pijuan, Tiziano A. Schweizer, Srikanth Mairpady Shambat, Barbara Hasse, Annelies S. Zinkernagel, Silvio D. Brugger

**Affiliations:** a Department of Infectious Diseases and Hospital Epidemiology, University Hospital Zurich, University of Zurich, Zurich, Switzerland; Riverside University Health System, Medical Center/University of California

**Keywords:** *Staphylococcus aureus*, colonization, invasive infection, transition, barrier breach, fitness, genomics, upper respiratory tract

## Abstract

Staphylococcus aureus carriage is a risk factor for invasive infections. Unique genetic elements favoring the transition from colonizing to invasive phenotype have not yet been identified, and phenotypic adaptation traits are understudied. We therefore assessed phenotypic and genotypic profiles of 11 S. aureus isolate pairs sampled from colonized patients simultaneously suffering from invasive S. aureus infections. Ten out of 11 isolate pairs displayed the same *spa* and multilocus sequence type, suggesting colonization as an origin for the invasive infection. Systematic analysis of colonizing and invasive isolate pairs showed similar adherence, hemolysis, reproductive fitness properties, antibiotic tolerance, and virulence in a Galleria mellonella infection model, as well as minimal genetic differences. Our results provide insights into the similar phenotypes associated with limited adaptation between colonizing and invasive isolates. Disruption of the physical barriers of mucosa or skin was identified in the majority of patients, further emphasizing colonization as a major risk factor for invasive disease.

**IMPORTANCE** S. aureus is a major pathogen of humans, causing a wide range of diseases. The difficulty to develop a vaccine and antibiotic treatment failure warrant the exploration of novel treatment strategies. Asymptomatic colonization of the human nasal passages is a major risk factor for invasive disease, and decolonization procedures have been effective in preventing invasive infections. However, the transition of S. aureus from a benign colonizer of the nasal passages to a major pathogen is not well understood, and both host and bacterial properties have been discussed as being relevant for this behavioral change. We conducted a thorough investigation of patient-derived strain pairs reflecting colonizing and invasive isolates in a given patient. Although we identified limited genetic adaptation in certain strains, as well as slight differences in adherence capacity among colonizing and invasive isolates, our work suggests that barrier breaches are a key event in the disease continuum of S. aureus.

## INTRODUCTION

Staphylococcus aureus is a frequent asymptomatic colonizer of the human skin and mucosa and is identified as part of the normal human microbial flora (1). S. aureus carriage can be persistent (~20%) or intermittent (~60%) (2). Nevertheless, colonization puts the individual at increased risk for developing invasive infections, ranging from simple skin infections to life-threatening infections (e.g., from superficial wound infections and abscesses to bacteremia and endocarditis) (1, 3). S. aureus is therefore classified as a pathobiont, reflecting this dual behavior of a colonizer and a pathogen and the corresponding damage-response continuum, and is capable of infecting both healthy and immunocompromised individuals (4, 5).

In industrialized countries, the annual incidence rate for S. aureus bloodstream infection is 26.1 per 100,000 inhabitants, with a case-fatality rate of around 25% (6). Treatment of S. aureus infections faces various obstacles, such as antibiotic resistance, tolerance, and bacterial persistence (7). Given the burden of S. aureus disease, different prevention strategies are currently under investigation. Decolonization has proven efficient in preventing nosocomial S. aureus infections among carriers (3, 8).

The exact circumstances under which a colonizing S. aureus strain becomes invasive and causes host damage are incompletely understood. Invasive S. aureus infections most probably originate from the bacteria colonizing the nose and throat. A breach of the protective physical barriers of the skin and mucosa allows the bacteria to enter the bloodstream and spread to other body sites (9). Information supporting this hypothesis comes from studies comparing invasive and colonizing S. aureus strains isolated from the same patient where over 80% of isolate pairs were identical, as determined by pulsed-field gel electrophoresis (10, 11). Research carried out in this field compared genotypes and virulence gene expression among colonizing and invasive S. aureus strains, as well as factors influencing the transition (12–17). However, to our knowledge, a phenotypic and genotypic assessment of strain adaptation during transition from a colonizing to an invasive behavior by matching colonizing and invasive isolates derived from the same patient is still lacking. The aim of this study was therefore to analyze the genetic traits, virulence, reproductive fitness, and antibiotic tolerance capacity of corresponding colonizing and invasive S. aureus strains isolated from the same patient and carrying the same *spa* and multilocus sequence types (MLSTs) are referred to as isolate pairs.

## RESULTS

### Patient cohort and clinical isolates.

Thirty-eight patients suffering from distinct acute invasive S. aureus infections were enrolled in the BacVivo study between January and November 2019. Among these, 11 tested positive for S. aureus colonization in the nose or groin and were selected for this project (Table 1). Patients showed a variety of different S. aureus infections ranging from surgical wound infection to extensive skin infections and bacteremia. The patient cohort consisted of seven male and four female patients, with ages ranging between 29 and 86 years. The duration of infection before strain isolation spanned between zero and 15 days (average, 3.6 days). In seven patients, a barrier breach was associated with infection.

**TABLE 1:** Patient demographic data, infection types, and relevant comorbidities^*a*^

Patient	Age/sex	S. aureus infection	Duration of infection^*b*^	Comorbidities	Postulated entry point
1	68/M	Sepsis, septic arthritis of facet joints (L4/5, L5/S1), associated abscesses (epidural, paravertebral, *Musculus psoas*)	2/1	Renal cell carcinoma, CKD, T2DM, HTN, obesity	ND
2	45/M	Endocarditis, associated abscesses of *M. iliacus* and *M. piriformis*	2/1	Recurrent sinusitis	ND
3	83/M	Endocarditis and hemodialysis catheter infection	1/0	HFpEF, CKD, T2DM, biclonal gammopathy type IgM	Permcath jugular vein right
4	77/M	Superinfection of nasal tamponade, periorbital cellulitis, sepsis	2/2	HTN, T2DM	Recent epistaxis
5	65/M	Infection of left ventricular assist device, bacteremia	0/6	HFrEF, CHD, CKD, total thyroidectomy with thyroid hormone substitution	LVAD (01.2018)/CRT-P (12.2017)
6	31/F	Spondylodiscitis Th 10/11, bacteremia	5/2	Atopic diathesis, allergy to beta-lactam antibiotics	Heel wound, atopic diathesis with chronic urticaria
7	86/F	Subcutaneous abscess and osteomyelitis (surgical site infection)	3/3	Thigh fracture, atopic eczema, asthma, T2DM, obesity, osteoporosis	Atopic eczema
8	46/M	Surgical wound infection	1/4	Catheter-associated bacteremia with S. epidermidis, polytrauma	PICC-line
9	63/F	Extended soft tissue infection	8/1^*c*^	COPD, substituted hypothyroidism	ND
10	79/M	Foreign body-associated infection spondyloidesis (C1–C7), bacteremia, suspected abscess formation and CNS involvement	1/6	Lung carcinoma stage IV with cervical spine metastasis, malnutrition, HTN	Operation at the abscess site 3 months before infection
11	29/F	Perforated appendicitis with polymicrobial abscess formation	15/0		ND

a M, male; F, female; CNS, central nervous system; CKD, chronic kidney disease; T2DM, type 2 diabetes mellitus; HTN, hypertension; HFpEF, heart failure with preserved ejection fraction; HFrEF, heart failure with reduced ejection fraction; CHD, coronary heart disease; COPD, chronic obstructive pulmonary disease; LVAD, left ventricular assist device; CRT-P, cardio resynchronization therapy pacemaker; PICC-line, peripherally inserted central catheter line; ND, not determined; Th, thoracic vertebrae.

b That is, the duration of infection before strain isolation (days)/time between isolation of invasive and colonizing isolates (days).

c The colonizing isolate was isolated prior to the invasive isolate.

### Antibiotic susceptibility testing.

Of the 22 strains, the two collected from patient 8 were methicillin-resistant S. aureus (MRSA). Antibiotic susceptibility testing results are listed in Table 2 (see also Table S1 in the supplemental material).

**TABLE 2:** Clinical isolates information^*a*^

Patient	Strain	Origin	*spa* type	MLST	Antibiotic		MIC (μg/mL)	
					Therapy (days)	Resistance/MRSA	CIP (CLI)	FLU
1	CI1814	Blood	t3802	6099 (new)	None	BEN, AMP, ERY, CLI	0.25	0.25
	CI1818	Nose	t3802	6099 (new)	AMC (1), FLU (1)	BEN, AMP, ERY, CLI	0.25	0.25
2	CI2016	Blood	t359	97	FLU (1)	ND	0.25	0.25
	CI2025	Nose	t359	97	FLU (2), CLI (1)	BEN, AMP	0.25	0.25
3	CI2050	Blood	t116	45	PIT (1), VAN (1)	ND	0.25	0.25
	CI2056	Nose	t116	45	PIT (4), VAN (5), FLU (4)	ND	0.25	0.25
4	CI2182	Blood	t3982	8	None	ND	0.25	0.25
	CI2327	Nose	t3982	8	VAN (1), AMC (2)	ND	0.25	0.25
5	CI2647	Blood	t19435 (new)	88	CIP (1), CLA (1)	BEN, AMP	0.25	0.25
	CI2670	Nose	t19435 (new)	88	CIP (2), CLA (2), VAN (3), FLU (6)	BEN, AMP	0.25	0.25
6	CI3183	Blood	t127	6100 (new)	None	ND	0.25	0.25
	CI3203	Nose	t127	6100 (new)	VAN (2)	ND	0.25	0.25
7	CI3984	Tissue	t008	8	AMC (1)	ND	0.25	0.25
	CI3990	Nose	t008	8	AMC (3), FLU (2)	ND	0.25	0.25
8	CI4099	Tissue	t2237	22	PIT (17)	BEN, AMP, CXI, MOX, NOR/MRSA	(0.125)	0.25
	CI4130	Groin	t2237	22	PIT (21), VAN (14)*	BEN, AMP, CXI, MOX, NOR, CIP, LEV, SFX, PEF/MRSA	(0.125)	0.25
9	CI4090	Tissue	t665	1472	MER (2), VAN (2), GEN (2)	BEN, AMP, CXI, MOX, TOB, ERY	0.5	1.00
	CI4097	Groin	t665	1472	MER (4), VAN (10), GEN (4)	BEN, AMP, CXI, MOX, ERY	0.5	1.00
10	CI4116	Blood	t310	22	FLU (1)	BEN, AMP	0.5	0.25
	CI4152	Nose	t310	22	AMC (4), VAN (1), FLU (7), DAP (1)	BEN, AMP	0.5	0.25
11	CI4065	Tissue	t2078	101	AMC (2), MET (2)	BEN, AMP, ERY, CLI	0.25	0.25
	CI4077	Nose	t002	5	AMC (6), MET (6)	BEN, AMP	0.25	0.25

a Strain information, including strain ID, origin, *spa* (staphylococcal protein A) type and multilocus sequence type (MLST), antimicrobial therapy before isolation (in days), resistance to tested antibiotics, classification as methicillin-resistant S. aureus (MRSA) and MIC for ciprofloxacin (CIP), clindamycin (CLI), and flucloxacillin (FLU). Breakpoints can be found at EUCAST (45). ND, none detected (no antibiotic resistance detected for the tested antibiotics); AMC, amoxicillin-clavulanate; AMP, ampicillin; BEN, benzylpenicillin; CLA, clarithromycin; CXI, cefoxitin; DAP, daptomycin; ERY, erythromycin, GEN, gentamicin, LEV, levofloxacin, MER, meropenem, MET, metronidazole, MOX, moxifloxacin, NOR, norfloxacin, PEF, perfloxacin. PIT, piperacillin-tazobactam, TOB, tobramycin, SPX, sparfloxacin, VAN, vancomycin. * PICC (peripherally inserted central venous catheter) line infection with *S. epidermidis*.

### Strains *spa* and MLST characterization.

In order to assess potential clonality of the colonizing and invasive S. aureus isolates, all clinical isolates included in this study were initially *spa*-typed. Twelve different *spa* types were found (Table 2). In 10 of 11 cases, the invasive and the colonizing S. aureus strains showed the same *spa* and multilocus sequence type (MLST), and among the matching pairs one new *spa* type (t19435) and two new MLST profiles (ST-6099 and ST-6100) were found and submitted to PubMLST (https://pubmlst.org/).

### Whole-genome sequencing reveals minimal genomic evolution during transition from colonization to invasion.

To investigate genetic differences as a measure of genetic evolution during transition from colonization to invasive disease, whole-genome sequencing of all isolates was performed. The diversity of the isolates from patient 11 was confirmed, as illustrated by their distance on the core genome-based phylogenetic tree (see Fig. S1 in the supplemental material). All other isolate pairs clustered together, and a reference strain closely related to each pair was chosen for comparative genomics (see Fig. S1 and Table S2).

None of the 10 pairs of isolates with matching *spa* type and MLST displayed gene content differences. At the core genome level, 40% of them (4/10)—namely, CI3990-CI3984 (patient 7), CI4130-CI4099 (patient 8), CI4097-CI4090 (patient 9), and CI4152-CI4116 (patient 10)—were found to be identical. The remaining six pairs displayed between 1 and 26 core genome variants (insertion, deletions, and single nucleotide polymorphisms [SNPs]) (see Table S3). Of these six pairs, two—CI2670-CI2647 (patient 5) and CI3203-CI3203 (patient 6)—displayed only synonymous or outside-from-coding-sequence mutations. This was the case for 64% (51/80) of the total variants identified. The 29 nonsynonymous variants, affecting CI1418-CI1818 (patient 1), CI2016-CI2025 (patient 2), CI2056-CI2050 (patient 3), and CI2182-CI2327 (patient 4), are shown in Table 3. Most were missense variants (*n* = 23), and the remaining were one in-frame deletion, four frameshift variants, and one premature stop.

**TABLE 3:** Nonsynonymous mutations differentiating isolate pairs^*a*^

Isolate 1	Reference strain	Position	Type	Effect		Gene	Protein annotation	Product
				Nucleotide	Amino acid			
CI1814	NZ_CP047021	572249	SNP	128T>C	Leu43Ser		GQX63_RS02655	Amidohydrolase
CI1818	NZ_CP047021	1733476	SNP	156A>G	Ile52Met		GQX63_RS08235	HAD family hydrolase
CI1814	NZ_CP047021	2532505	SNP	688G>A	Val230Ile		GQX63_RS12650	Membrane protein
CI1818	NZ_CP047021	2474835	SNP	359T>A	Leu120*		GQX63_RS12355	ABC transporter ATP-binding protein
CI1818	NZ_CP047021	1430782	Del	13886delG	Gly4629fs	*ebh*	GQX63_RS06790	Hyperosmolarity resistance protein Ebh
CI1814	NZ_CP047021	2027060	SNP	1835A>G	Asp612Gly		GQX63_RS09955	Hypothetical protein
CI1818	NZ_CP047021	2080129	SNP	283T>C	Tyr95His	*agrA*	GQX63_RS10315	Response regulator transcription factor
CI1818	NZ_CP047021	2172106	SNP	113A>G	Gln38Arg		GQX63_RS10785	UDP-*N*-acetylglucosamine 1-carboxyvinyltransferase
CI2016	NZ_CP029629	2121258	SNP	566C>T	Thr189Ile		DLJ56_RS10985	DMT family transporter
CI2025	NZ_CP029629	295778	SNP	1199C>T	Thr400Ile		DLJ56_RS01315	Ribonuclease YeeF family protein
CI2025	NZ_CP029629	929336	Del	36delT	Phe12fs		DLJ56_RS05000	Hypothetical protein
CI2025	NZ_CP029629	2435974	Del	915_926del TGCAATTGGTGG	Ala306_ Gly309del		DLJ56_RS12685	PTS sucrose transporter subunit IIBC
CI2025	NZ_CP029629	796734	SNP	167A>G	Gln56Arg	*gatA*	DLJ56_RS04105	Asp-tRNA(Asn)/Glu-tRNA(Gln) amidotransferase subunit GatA
CI2016	NZ_CP029629	17890	SNP	85T>C	Phe29Leu	*gdpP*	DLJ56_RS00075	Cyclic-di-AMP phosphodiesterase GdpP
CI2025	NZ_CP029629	2321375	SNP	766T>C	Ser256Pro		DLJ56_RS12045	Efflux RND transporter permease subunit
CI2016	NZ_CP029629	471537	SNP	128G>C	Arg43Pro		DLJ56_RS02390	Hypothetical protein
CI2056	NZ_CP014791	985658	SNP	406G>T	Gly136Cys		CKU_RS04795	Phosphoribosylformylglycinamidine cyclo-ligase
CI2056	NZ_CP014791	718203	Del	105_117del CTTACCAACTGGT	Leu36fs	*recQ*	CKU_RS03475	DNA helicase RecQ
CI2056	NZ_CP014791	1173973	SNP	889G>A	Gly297Ser		CKU_RS05740	Methylenetetrahydrofolate−tRNA-[uracil(54)-C(5)]-methyltransferase [FADH(2)-oxidizing] TrmFO
CI2327	CP000255	1024038	SNP	572C>T	Ala191Val		SAUSA300_0934	Membrane protein
CI2182	CP000255	1263673	SNP	200C>T	Ser67Phe	*frr*	SAUSA300_1152	Ribosome recycling factor
CI2182	CP000255	1789880	SNP	731G>A	Arg244His	*mutM*	SAUSA300_1635	Formamidopyrimidine-DNA glycosylase
CI2327	CP000255	1651324	SNP	271A>G	Ile91Val		SAUSA300_1497	Glycine dehydrogenase, subunit 1 (glycine cleavage system P protein)
CI2327	CP000255	1509350	SNP	11A>C	Tyr4Ser		SAUSA300_1344	Putative DNA replication protein DnaD
CI2327	CP000255	194570	SNP	500C>T	Ala167Val		SAUSA300_0171	Cation efflux family protein
CI2182	CP000255	402701	SNP	203A>G	Glu68Gly		SAUSA300_0352	ABC transporter, ATP-binding protein
CI2327	CP000255	394277	SNP	461G>T	Ser154Ile		SAUSA300_0341	Putative membrane protein
CI2327	CP000255	1880776	Ins	3692dupA	Asn1231fs		SAUSA300_1702	Cell wall surface anchor family protein
CI2182	CP000255	2195295	SNP	209G>T	Gly70Val	*kdpB*	SAUSA300_2033	K^+^-transporting ATPase, B subunit

a This is a subset of Table S3 in the supplemental material, which includes all mutations differentiating isolate pairs. Here, only nonsynonymous mutations are shown, since they might affect protein functions and thus have a biological impact. The changes are given with respect to publicly available assembled references, whose GenBank accession numbers are provided in the second column. The base pair position, mutation type, and effect at the nucleotide/amino acid level, as well as the annotation and product of the affected proteins in the corresponding reference genome, are shown. For each pair, a closely related reference was chosen. Importantly, note that the original allele/protein variant of the common ancestor of the two isolates is unknown. SNP, single nucleotide polymorphism; Del, deletion; Ins, insertion; *, premature stop codon; fs, frameshift; nt, nucleotide, aa, amino acid.

Among other mutations, a missense variant (Tyr95His) of *agrA* distinguished CI1818 from CI1814. Within-host mutations of this master regulator gene of S. aureus virulence have been previously described and are expected to be phenotypically relevant. In addition, a frameshift variant (Gly4629fs) of Ebh, the extracellular matrix-binding protein might result in altered adherence capacity, complement resistance, and pathogenesis during infection (18–21). Among the variants distinguishing CI2016 from CI2025, a missense variant of GdpP could be phenotypically relevant. Finally, a missense variant (Arg244His) of MutM, a protein involved in DNA mismatch repair, distinguished CI2182 from CI2327. An increased mutation rate would be a plausible explanation for the substantial diversification of this strain pair compared to others: the 26 variants between these two isolates were the highest genetic distance across pairs. In conclusion, the clonal origin of the isolate pairs was confirmed, and different extents of diversification could be observed in different patients. Phenotypic analysis was carried out on the 10 isolate pairs displaying the same *spa* type and MLST.

### Whole-genome analysis using long reads confirms that the appearance of differentially located insertion sequences does not take place during transition from colonization to invasion.

It is well known that insertion elements are associated with phenotypic variation in S. aureus strains, and some of them (e.g., IS*256*) have even been described as potential drivers of microevolution (13). In order to investigate whether transposable elements appear during the transition from colonization into invasive bacterial infections, we have performed long-read sequencing of the 22 clinical isolates.

Comparison of the insertion sequences (ISs) of 11 isolate pairs revealed that none of the 10 pairs with matching *spa* type and MLST acquired any differentially located ISs (DLIS). In contrast, several ISs were found at different locations in the strains CI4065 and CI4077, displaying a different *spa* type (patient 11), in agreement with the previously described genetic diversity of these two isolates. Outputs of ISCompare are shown in Table S4.

### Comparable hemolysis, adherence properties, and reproductive fitness of colonizing and invasive *S. aureus* isolate pairs.

**FIG 1:**
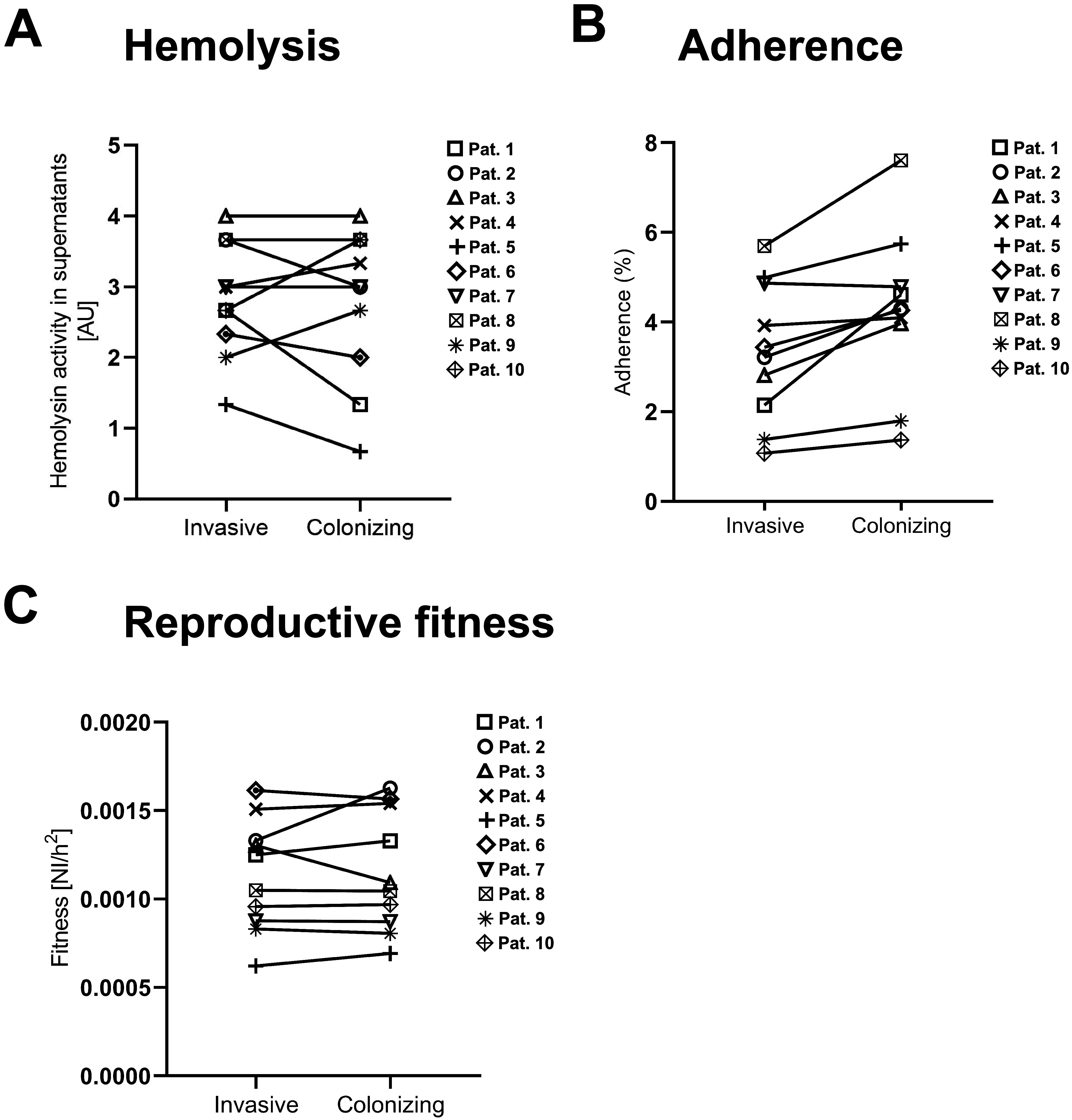
Comparable hemolysis, adherence properties, and reproductive fitness of colonizing and invasive S. aureus strains and strain pairs. (A) Hemolysis activity detected in S. aureus clinical isolates supernatants. Serial dilutions (1:2) of supernatants derived from OD_600_ 6 overnight cultures of the strains of interest were incubated with human erythrocytes for 1 h at 37°C, followed by 30 min at 4°C. The absorbance at 415 nm was measured to detect hemolysis, and the last dilution displaying activity was plotted on the graph. Each data point displays the mean value of at least three independent experiments. (B) Adherence of S. aureus *clinical* isolates to human adenocarcinoma human lung alveolar basal epithelial cells (A549). Mid-logarithmic-growth-phase bacteria were added to A549 cells at an MOI of 10. After incubation at 37°C in 5% CO_2_ for 30 min, the cells were washed with PBS and lysed with PBS + 0.02% Triton X-100, and adhering bacteria were then enumerated by plating serial dilutions of this suspension. Each data point displays the mean value of at least three independent experiments. (C) Reproductive fitness of invasive and colonizing S. aureus clinical isolates. The reproductive fitness was calculated according to the Gompertz mathematical model as the ratio between the MGR and time to reach MGR (*T*_max_) displayed by the various isolates. NI/h^2^, normalized intensity per square hour; Pat, patient. Each data point displays the mean value of at least three independent experiments. Statistical significance was assessed using the paired *t* test on the average values of invasive versus colonizing strains. Average hemolysis (invasive versus colonizing), 2.833 AU versus 2.733 AU (*P* = 0.6557); average adherence (invasive versus colonizing), 3.355% versus 4.253% (*P* = 0.0059); average reproductive fitness (invasive versus colonizing), 0.0011 NI/h^2^ versus 0.0012 NI/h^2^ (*P* = 0.6311).

We next characterized phenotypically the 10 pairs of isogenic isolates with the same *spa* type and MLST. The hemolytic capability of the S. aureus clinical isolates was tested. However, no significant difference was found across strains or between invasive and colonizing isolates (hemolysis average, 2.833 AU versus 2.733 AU; *P* = 0.6557 [with paired *t* test]) (Fig. 1A). Since adherence to host cells is the first step leading to invasion and spread of a bacterial infection, we compared the adherence capacity of invasive versus colonizing isolates. Although adherence to the lung epithelial cell line A549 showed significant difference between invasive and colonizing isolates (adherence average, 4.253% versus 3.335%; *P* = 0.0059 [with paired *t* test]), the biological relevance of this difference is debatable considering the low rates of invasion and the limited sample size (Fig. 1B). To compare the growth characteristics and reproductive fitness, quantitative fitness analysis (QFA) was performed. Invasive versus colonizing isolates showed no difference in reproductive fitness (0.0011 normalized intensity per square hour [NI/h^2^] versus 0.0012 NI/h^2^, *P* = 0.6311 [with paired *t* test]) (Fig. 1C).

### Persisters formation and colony size distribution do not vary between colonizing and invasive *S. aureus* isolates.

**FIG 2:**
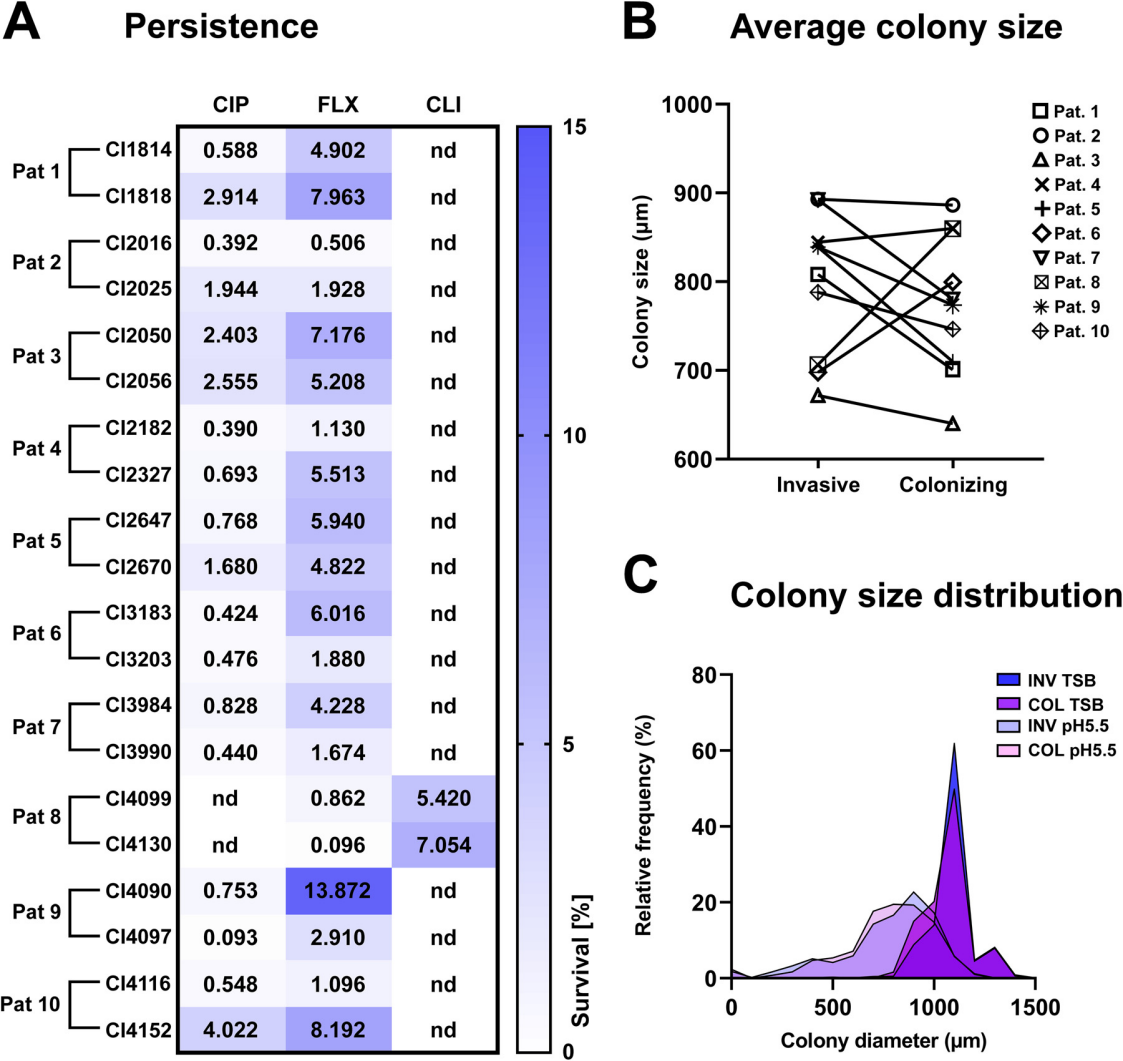
Persister formation and colony size distribution do not vary between colonizing and invasive S. aureus strains and strain pairs. (A) Persister formation was assessed on S. aureus strains after incubation in pH 5.5 medium for 72 h at 37°C in 5% CO_2_, followed by incubation for 24 h in pH 7.4 medium in the presence or absence of 40× the MIC of either ciprofloxacin (CIP), flucloxacillin (FLX), or—if ciprofloxacin resistant—clindamycin (CLI). After 24 h, the antibiotics were washed out, and the surviving bacteria were plated on TSB plates and counted. The figure shows the surviving bacteria as a percentage of the inoculum. At least four biological replicates were carried out per strain. The paired *t* test was used to determine statistical significance between the colonizing and invasive strains. (B) The average colony size was assessed for invasive and colonizing strains by measuring the colony size of the S. aureus clinical isolates after 72 h of pH 5.5 stress and subsequent incubation at 37°C for 24 h on blood agar plates. Each data point displays the mean value of at least three independent experiments. Statistical significance was assessed using a paired *t* test on the average values of invasive versus colonizing strains. Average colony size (invasive versus colonizing), 798.037 μm versus 775.689 μm (*P* = 0.4643). (C) Colony size distribution was assessed for invasive and colonizing strains by measuring the colony size of the S. aureus clinical isolates after 72 h of pH 5.5 stress or 72 h of growth in TSB and subsequent incubation at 37°C for 24 h on blood agar plates. A minimum of three biological replicates were carried out per strain. Pat, patient; INV, invasive; COL, colonizing; nd, not determined.

In order to evaluate the phenotypic plasticity of the 10 pairs of isogenic isolates with the same *spa* type and MLST upon environmental stress, we assessed their baseline rate of persisters formation and their ability to form persisters upon acidic stress *in vitro*. The baseline rate of persisters formation did not differ greatly among the isolates (see Fig. S2). After pH stress exposure, the clinical isolates were challenged with antibiotics. Generally, the different strains responded in a very similar manner to pH stress, followed by antibiotic challenge (Fig. 2A).

The persisters triggered by pH stress need some time to exit their dormancy state, which results in nonstable small colonies (nsSCs) upon plating on solid nutrient medium. nsSCs can be detected from a bacterial population and are an index of the presence of persisters (22–24). Hence, we also characterized the colony size heterogeneity of bacteria upon pH challenge compared to bacteria grown in rich medium (tryptic soy broth [TSB]). No significant differences in average colony size were found across invasive and colonizing isolates (colony size average, 798.037 μm versus 775.689 μm; *P* = 0.5190) or between isolate pairs after pH stress (Fig. 2B). A general shift in colony size distributions, with long tails of small colonies, was observed upon pH stress compared to growth in rich medium (Fig. 2C). This was independent of the strain’s origin being either colonizing or invasive.

### Similar virulence of colonizing and invasive *S. aureus* isolates *in vivo*.

**FIG 3:**
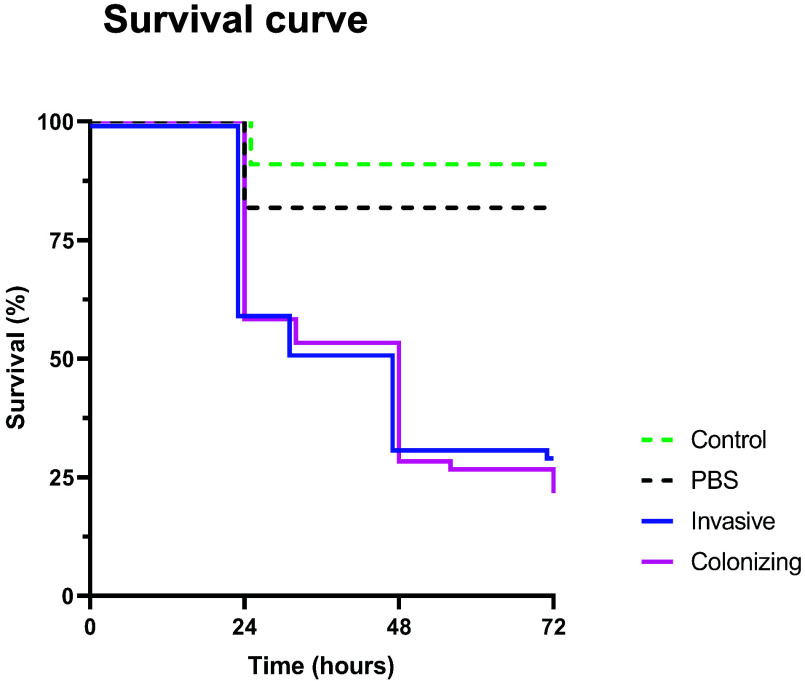
Effect of S. aureus clinical isolate strains on the viability of G. mellonella larvae over 72 h. G. mellonella was infected with 10^4^ CFU in 10 μL of PBS. Afterward, G. mellonella was incubated at 37°C, and the viability was assessed over a 72-h period. Each test group contained six Galleria mellonella, and the graph shows the results of one experiment. Statistical differences between the survival curves were assessed using the log-rank test. Invasive versus colonizing, *P* = 0.4146; invasive versus PBS control, *P* = 0.0042; colonizing versus PBS control, *P* = 0.0005.

G. mellonella larvae were used to investigate the virulence of the S. aureus clinical isolates *in vivo*. Larvae injected with colonizing or invasive S. aureus isolates showed a significantly decreased survival compared to phosphate-buffered saline (PBS)-injected ones (invasive isolates, *P* = 0.0023; colonizing isolates, *P* = 0.0002), and no difference between colonizing and invasive isolates was found (*P* = 0.3541) (Fig. 3). There was no significant difference between the survival of PBS-injected and noninjected larvae.

## DISCUSSION

We characterized 10 colonizing and invasive S. aureus isolate pairs from patients with acute invasive S. aureus infections and found a similar phenotype associated with limited adaptive genetic evolution between the colonizing and the invasive isolates. Breaching of the skin or mucosa was found in most of the patients, underlining the observation that colonization is a major risk factor for S. aureus invasive infections. Thus far, various studies have assessed genotypic characterization but failed to pinpoint traits responsible for the transition from colonizing to invasive behavior. Aiming to assess whether phenotypic properties would influence the invasion characteristics of the isolated strains, extensive analysis of virulence traits of S. aureus isolate pairs was carried out using well-established *in vitro* and *in vivo* models coupled with techniques for the detection of S. aureus fitness and persisters. Despite the broadness of approaches, no significant differences in virulence or environmental stress adaptation could be detected, mirroring results obtained previously (16), calling for a more in-depth analysis of the role of host factors in the transition between colonizing and invasive phenotypes. Future studies should focus on strains isolated from cohorts of specific S. aureus diseases, such as skin and soft tissue infections, bacteremia, endocarditis, etc., to better define adaptation in a specific disease contest. As a first step, we screened the invasive and colonizing clinical isolates for their genetic background and antibiotic susceptibility spectrum. We found that the isolate pairs shared, in 10 of 11 cases, the same *spa* type and MLST, thus strengthening the hypothesis that colonizing S. aureus strains are the reservoir from which invasive infections originate. The genetic concordance between colonizing and invasive strains isolated from the same individual was shown before (10, 11, 25), confirming a common pattern.

To assess virulence, fitness and persisters formation of invasive and colonizing isolates, we decided to limit the investigation to the 10 strain pairs presenting the same *spa* type and MLST profiles and excluded the two strains derived from patient 11.

To compare the virulence of our panel of clinical isolates, we investigated the role of two key virulence factors in the pathology of S. aureus infections: hemolysis and adherence to eukaryotic cells. The pore-forming toxin α-hemolysin, also known as alpha-toxin, is a main virulence factor of S. aureus influencing hemolysis (26, 27), and its activity was measured for colonizing and invasive clinical isolates. Our results show that colonizing strains do not differ from invasive strains in their capacity to lyse red blood cells under the conditions tested using our experimental settings. Despite differences in hemolysis levels found among invasive as well as colonizing strains, spanning from 1 to 4 AU (where 1 AU represents one 1:2 dilution series), the overall data show no significant difference between the two groups.

The ability to adhere to and invade epithelial cells plays a major role in S. aureus colonization, both biofilm formation and early manifestation of S. aureus infection, and was previously shown to vary among different S. aureus strains (28). Furthermore, irrespective of the location, adherence increases the chance of the bacterium to breach defensive barriers (29). We tested the adherence of the S. aureus clinical isolates to lung epithelial cells. A significantly higher adherence was observed for colonizing strains. This might indicate a phenotypic alteration such as capsule expression or regulation of adherence factors. Nevertheless, the biological relevance of this difference remains uncertain given the low adherence level of the strains to host cells overall and the small sample size (30). A more comprehensive study including a higher number of isolates is needed to confirm this observation. Comparison of growth dynamics of the various clinical isolates allowed screening for changes in reproductive fitness possibly linked to the transition toward an invasive phenotype. Overall, we found a large variation in reproductive fitness among the S. aureus isolates. However, although the fact that we did not observe differences between isolate pairs or between colonizing versus invasive isolates might indicate that reproductive fitness does not play a role in the transition from colonizing to invasive phenotype for the strains analyzed in this work. There remains the possibility that the fitness might be greater compared to that of a common ancestor that evolved while colonizing.

Survival in the host is a key factor that allows bacterial spread during an invasive infection. Exposure of bacteria to environmental stress, such as low pH or residing in the host, leads to the formation of a subpopulation of persister cells (22, 23, 31) characterized by a growth-arrest phenotype that allows them to survive both host defense mechanisms and antibiotic treatment targeting active cellular processes. We hypothesized that invasive isolates may display a higher degree of persister cell formation due to the exposure to host environmental stressors such as pH, antibiotics, and immune cells. We therefore examined the capability of colonizing and invasive S. aureus isolates to form a subpopulation of persister cells upon pH stress and subsequent antibiotic exposure. The survival of invasive and colonizing clinical isolates did not differ, with all strains displaying a very similar survival profile. This suggests that the ability to form persisters upon pH stress is similar for both colonizing and invasive isolates. Persisters, as a result of their slow-growth phenotype, generate phenotypically heterogeneous nsSCs (22). As an estimate of the presence of a persister population, we also compared the average colony size and colony size distribution of colonizing and invasive clinical isolates after pH stress. No difference in average colony size was found, indicating a similar capacity to form a persister population in both sets of isolates. Colony size distribution plots, in which we compared the colony size of strains grown in nutrient-rich medium and neutral pH with the colony size of strains grown in nutrient-poor medium under pH stress conditions, indicate the presence of a consistent subpopulation of persisters in all clinical isolates.

Next, we tested the virulence of the clinical isolates *in vivo*, using G. mellonella larvae as a model organism (32). G. mellonella larvae provide a cost-effective, easy-to-handle, and robust animal model to study S. aureus infection in a complex organism, more closely mimicking an infection in the human host then *in vitro* models (33–37). Colonizing and invasive clinical isolates were compared for their ability to infect and kill the larvae. No survival differences were found either between the two groups of isolates or between strain pairs.

Whole-genome sequence analysis confirmed the absence of specific determinants causing the transition to a more virulent phenotype, a finding in line with previous work showing similar gene content between invasive and colonizing isolates (38). We identified mutations differentiating pairs of isolates in 6 of the 10 cases examined. Moreover, only one isolate per niche was obtained, and the spatiotemporal dynamics of the accumulation of the detected mutations are unknown. None of the identified mutations recurred in multiple cases. Four of the ten isolate pairs were genetically identical, while few mutations distinguished the remaining six pairs. Of these, none recurred in several pairs. For the six strain pairs with variants, nonsynonymous mutations were only observed in four strain pairs, and mutations were detected in both colonizing and invasive strains compared to the reference strain. Although most phenotypic differences were not statistically significant, this cannot rule out an *in vivo* benefit of certain mutations (39). Since the insertion element IS*256* has been identified as a possible driver of within-host microevolution in selected lineages, we additionally performed long-read sequencing (13). However, hybrid assemblies showed no structural rearrangements or movement of IS elements between colonizing and invasive strains belonging to the same isolate pair.

Previous works carried out on S. aureus isolates also showed equal occurrence of virulence genes among S. aureus colonizing and invasive isolates, mirroring our results on larger sample cohorts (14, 16, 38, 40). However, some isolates contained mutations that might reflect adaption, and other groups have reported mutations associated with the transition from colonization to invasive disease or during invasive disease (12, 41). Young et al. (42) analyzed three S. aureus carriers longitudinally and found only eight mutations accompanied the transition of an asymptomatically carried MSSA population to a fatal bloodstream infection, with half of those mutations being premature stop codons. Abu Othman et al. also tested the virulence gene expression of S. aureus clinical isolates during growth in the presence of human serum to better mimic host conditions (17). A high variation in gene expression among different clinical isolates was observed, and the expression of the collagen adhesion-encoding gene *cna* was upregulated in colonizing isolates, while the γ-hemolysin Hlg was upregulated in invasive isolates, confirming that the environmental conditions can influence strain behavior (17). Another interesting study showed the importance of environmental changes for virulence determinant expression by comparing S. aureus virulence factor expression in animal models of colonization, bacteremia, and endocarditis, highlighting the increased expression of a set of genes as important for the transition from colonizing to invasive behavior (15).

In conclusion, we examined several genetic and phenotypic characteristics that confer virulence to S. aureus to elucidate the main players involved in the transition from colonizing to invasive behavior. The absence of significant differences in virulence between invasive and colonizing strains we observed here does not reflect the disease progression observed in patients, where colonizing strains invaded and spread in the host, causing severe manifestations. The lack of differences between colonizing and invasive isolates are attributable to environmental and host factors such as breaching of the physical barrier. This is underlined by previous work which also suggests the likelihood of a host factor-mediated transition (38, 43). Since colonization is the preponderant risk factor for invasive staphylococcal disease and given the limited success of decolonization procedures, novel approaches such as decolonization of S. aureus with transplantation of beneficial bacterial commensals might be an attractive and efficient way to prevent invasive diseases.

## MATERIALS AND METHODS

### Patient recruitment.

The study was conducted as part of the BacVivo Bacterial Pathogen Properties in Patient Samples study, a single-center observational study conducted at the University Hospital of Zurich (44). This study was approved by local authorities (Cantonal Ethics Committee, Canton of Zurich, ethical application BASEC 2017-02225). Patients diagnosed with acute invasive S. aureus infection were screened for the presence of colonizing S. aureus in the nose and groin during their hospital stay. Patients with colonizing S. aureus were included in this analysis (Table 1). All patients included in this study signed an informed consent. Healthy volunteer blood was collected under protocol BASEC 2019-01735, approved by the Cantonal Ethics Committee, Canton of Zurich.

### *S. aureus* clinical isolates and processing of clinical samples.

S. aureus strains were isolated from either blood, tissue, nose, or groin (Table 2). Blood was cultured in BacT/Alert FA bottles (bioMérieux) at 37°C to positivity or up to 6 days. Tissue samples were homogenized using a mortar. Samples from homogenized tissues and swabs were cultured in thioglycolate broth to positivity. Antibiotic susceptibility testing (see Table S1) was carried out on Mueller-Hinton agar by using disk diffusion, and EUCAST breakpoints were used to interpret the results (45).

### *spa* typing.

*spa* typing was carried out as previously described (46). In brief, S. aureus was freshly grown on Columbia agar plus 5% sheep blood plates (bioMérieux). One colony of each strain was resuspended in 50 μL of nuclease-free water and microwaved for 2 min. Next, 1 μL of the resulting suspension was used for PCR. DreamTaq DNA polymerase (thermo Scientific) was used for amplification, together with spa-1113f (5′-TAA AGA CGA TCC TTC GGT GAG C-3′) and spa-1514r (5′-CAG CAG TAG TGC CGT TTG CTT-3′) primers. DNA purification was performed using a Wizard SV Gel and PCR Clean-Up System (Promega). Sanger sequencing was performed by Microsynth AG. The Ridom SpaServer program was used to analyze *spa* sequences, assign *spa* types, and calculate *spa* type frequencies (Ridom Spa Server).

### Whole-genome sequencing and comparative genomics using short reads.

DNA libraries were prepared with the QIASeq FX DNA library kit (Qiagen) and sequenced with a MiSeq reagent kit V2 (Illumina) on a MiSeq instrument. The 150-bp paired-end read files were cleared from Illumina adaptors and low-quality reads with Trimmomatic v.0.39 (47). *De novo* assemblies were built with SPAdes v.3.10 with the –careful command and the default k-mer size (48). The contigs with <2-fold average k-mer coverage and <200 bp were discarded. For MLST determination, the assemblies were scanned against traditional PubMLST typing schemes (https://pubmlst.org/) using MLST v.2.7.6 (GitHub). Annotation of our assemblies and the assemblies of selected reference genomes (see Table S2) was performed using Prokka v1.13 (49). The GFF files generated by Prokka were used as input to Roary v3.12 for pangenome construction (50). The command -e –mafft was used to generate a multi-fasta alignment of the core genes. Fasttree v2.1.10 (51) was used to build a maximum likelihood tree, with the default Juke Cantor model of nucleotide evolution. Variant calling between pairs of isolates was performed with Snippy v4.4.3 (52), by aligning the matching pair trimmed reads to the *de novo* assembly of one of the isolates, as well as to the .gbk file of the closest reference, to assess the effects of variants. Illumina paired-end reads of the 22 isolates are available under European Nucleotide Archive project PRJEB47806.

### Library preparation and sequencing for long reads.

The S. aureus clinical isolates were grown from single colony in 3 mL of TSB overnight at 37°C shaking. The cultures were subsequently diluted 1:10 in 3 mL of fresh TSB and grown for 2 h at 37°C with shaking. Then, 2 mL of the exponential-phase cultures were spun down at maximum speed for 1 min, and the bacterial pellet was resuspended in enzymatic lysis buffer (DNeasy Blood & Tissue kit; Qiagen) containing 20 mg/mL lysozyme and 100 μg/mL lysostaphin, followed by incubation for 30 min at 37°C. After the addition of proteinase K and buffer AL (DNeasy Blood & Tissue kit; Qiagen), the samples were incubated for 30 min at 56°C, and the samples were processed according to the manufacturer’s instructions.

The SMRTbell library was produced using SMRTbell Prep kit 3.0 (Pacific Biosciences). The input concentration was measured using a Qubit Fluorometer dsDNA high-sensitivity assay (Life Technologies). To assess the fragment size distribution, the samples were run on a Femto Pulse Device (Agilent). Approximately 1 μg of gDNA was mechanically sheared to an average size distribution of 7 to 10 kb, using a Megaruptor 3 Device (Diagenode). Then, 400 ng of sheared gDNA was DNA damage repaired and end repaired using polishing enzymes. A ligation was performed to create the SMRTbell template according to the manufacturer’s instructions. The final libraries were quality inspected and quantified using a Femto Pulse Device (Agilent) and a Qubit Fluorometer dsDNA high-sensitivity assay (Life Technologies). A ready-to-sequence SMRTbell-polymerase complex was created using a Sequel Binding kit 3.2 and Internal Control 1.0 (Pacific Biosciences) according to the manufacturer’s instructions. The Pacific Biosciences Sequel IIe instrument was programmed to sequence the libraries on one SMRT Cell 8M (PacBio) in HiFi mode, with a movie collection time of 15 h, using a Sequel II Sequencing kit 2.0 (Pacific Biosciences).

### Whole-genome sequencing and analysis of transposable elements using long reads.

DNA samples were also sequenced using Pacific BioSciences single-molecule real-time technology with a Sequel II System (PacBio SMRT Sequel IIe). The long reads were filtered by quality using Filtlong v0.2.1 (https://github.com/rrwick/Filtlong) and later assembled using Flye 2.9.1 (53). The resulting assemblies were polished using our previously described short reads with Polypolish v0.5.0 (54). Annotation of the hybrid assemblies was performed with Prokka v.1.13 (49), which generated GenBank files used as input to ISCompare 1.0.3 (55). ISCompare provided information on the differentially located insertion sequences between each pair of colonizing and invasive clinical isolates. PacBio SMRT reads of the 22 isolates are available through the European Nucleotide Archive project PRJEB47806.

### Hemolysis assay.

Hemolysis capacity of the various S. aureus strains was determined as previously described (31, 56), with minor changes. Clinical isolates were grown overnight in Todd-Hewitt broth (THB; BD). After adjusting the optical density at 600 nm (OD_600_) to 6, the overnight cultures were pelleted at 4,000 rpm for 10 min, and supernatants were filtered through a 0.22-μm-pore-size filter membrane (TPP). Human erythrocytes drawn from healthy volunteers were separated from plasma by centrifugation and washed three times with 150 mM NaCl and once with PBS. The erythrocytes were then diluted to a 5% suspension in PBS. Next, 50-μL portions of the resulting suspensions were added to 100 μL of 1:2 serial dilutions of the supernatants, followed by incubation for 1 h at 37°C, followed in turn by 30 min at 4°C. The plates were then centrifuged at 1,600 rpm for 5 min. Finally, 100 μL of supernatant was transferred to a new plate, and the absorbance was measured at 415 nm. The last supernatant dilution still showing hemolysis is depicted in the graph (Fig. 1A).

### Adherence assay.

Adherence of the S. aureus clinical isolates to human lung alveolar basal epithelial cells (A549) was tested, as previously described (57). A549 cells were cultured in Dulbecco modified Eagle medium (DMEM; Gibco) supplemented with 4.5 g/L d-glucose, l-glutamine, and 10% fetal bovine serum (FBS; Corning). For the assay, 10^5^ cells were seeded per well into 24 wells plates 36 to 48h prior to the assay. The S. aureus strains were grown overnight in TSB (BD), diluted 1:10 in 5 mL of fresh medium, and grown for 2 h to reach exponential growth phase. Bacteria were resuspended in DMEM supplemented with 2% FBS and then added to the cells at a multiplicity of infection (MOI) of 10. Plates were centrifuged at 1,200 rpm for 5 min and incubated at 37°C in 5% CO_2_ for 30 min. After incubation, the cells were washed three times with PBS; then, 500 μL of PBS containing 0.02% Triton X-100 were added to each well to lyse the eukaryotic cells. Serial dilutions of this suspension were plated on TSB agar plates for enumeration of adhering bacteria. To estimate the bacterial inoculum, 10 fold serial dilutions of the bacterial suspension used for infection were plated on TSB agar plates. Adherence was calculated as the percentage of recovered bacteria compared to the inoculum used for infection.

### Quantitative fitness analysis.

To compare the reproductive fitness of the clinical isolates, bacterial quantitative fitness analysis (BaQFA) was carried out as previously described (58). In brief, S. aureus strains freshly grown on TSB agar plates were harvested, resuspended in PBS to an OD_600_ of 0.1, and subsequently diluted 1:40 in PBS. A 96-pin replica plater was used to spot the bacterial suspension on a BHI (brain heart infusion; BD) agar plate, which was subsequently incubated at 37°C. Pictures of the plate were taken every 30 min using an automated setup. Optical density values were derived from the resulting time-lapse image series and fitted into a mathematical (Gompertz) growth model. A total of 72 spots per strain (24 spots of three biological replicates) were used for BaQFA calculations. For each strain, the maximal growth rate (MGR) and the time to reach MGR (*T*_max_) were measured, and the reproductive fitness was calculated as the ratio between MGR and *T*_max_ and expressed as the normalized intensity per square-hour (NI/h^2^).

### MIC.

The MICs for the antibiotics ciprofloxacin, clindamycin, and flucloxacillin were tested according to the broth microdilution method, following the recommendations of the European Committee on Antimicrobial Susceptibility Testing of the European Society of Clinical Microbiology and Infectious Disease (59). MICs were tested in pH 7.4 medium (35 mL of DMEM + 10% FBS +1% l-glutamine mixed with 12.62 mL of H_2_O and 2.38 mL of 1 M HEPES buffer).

### Persister assay.

To assess the fraction of persisters in the S. aureus clinical isolates, an assay based on colony size distribution and bacterial survival to high antibiotics concentration (40× MIC) upon low pH stress was conducted as previously described (22, 23). The strains were freshly grown on Columbia agar plus 5% sheep blood plates (bioMérieux) and afterward inoculated into filtered pH 5.5 medium (DMEM + 10% FBS + 1% l-glutamine mixed in a 7:3 ratio with pH 4 buffer [77.22 mM Na_2_HPO_4_ + 61.72 mM citric acid]) to an OD_600_ of 0.2. Afterward, bacteria were incubated for 72 h at 37°C in 5% CO_2_. 10 fold serial dilutions of the cultures were plated on Columbia agar plus 5% sheep blood plates for assessment of colony size distribution. Bacteria were subsequently diluted to 2 × 10^5^ CFU/mL into filtered pH 7.4 medium (35 mL of DMEM + 10% FBS + 1% l-glutamine mixed with 12.62 mL of H_2_O and 2.38 mL of 1 M HEPES buffer) in the presence or absence of 40× the MIC of the antibiotics ciprofloxacin, flucloxacillin, or clindamycin. The inoculum was estimated by plating serial dilutions of the no-antibiotic control culture. The bacteria were incubated for 24 h at 37°C in 5% CO_2_. The surviving bacteria were enumerated by plating 10 fold serial dilutions on TSB agar plates. The fraction of persisters in the total population was calculated as a percentage relative to the inoculum. To assess persister formation in logarithmic-growth-phase bacteria, S. aureus strains were grown in TSB overnight, diluted 1:10 in fresh TSB, and grown for 2 h. Bacteria were then incubated in the presence of 40× the MIC of flucloxacillin in pH 7.4 medium for 24 h at 37°C in 5% CO_2_ at an inoculum of 2 × 10^5^ CFU/mL. Persister levels were measured as described above.

To assess the colony size distribution after pH stress, bacteria recovered after 72 h of pH 5.5 exposure were plated on blood plates. To determine the average colony size for a bacterial population grown in rich medium, bacteria were grown overnight in TSB, diluted 1:10 in fresh medium, grown for 2 h, and subsequently plated on blood plates. Colony sizes were assessed after 24 h of incubation at 37°C. The plates were imaged with a Canon camera placed in a custom-built imaging box, and images were analyzed with ColTapp (60).

### *G. mellonella* virulence assay.

To study S. aureus virulence *in vivo*, the nonmammalian G. mellonella model system was used (61). The assay was conducted as previously described with minor modifications (56). G. mellonella in the final instar larval stage were purchased from HRH Hebeisen (Zurich, Switzerland) and stored at 4°C. S. aureus was grown overnight in TSB, diluted 1:10 in fresh TSB, and grown for another 2 h. Afterward, the bacteria were washed twice in PBS and then resuspended in PBS. Six G. mellonella per group were injected with 10^4^ CFU in a final volume of 10 μL into the last prolegs, either left or right, using a repetitive dispensing Tridak Stepper (Intertronic, Oxfordshire, England) with a 26-gauge needle syringe. A control group was injected with 10 μL of PBS, and a second control group of noninjected G. mellonella was also used. The larvae were subsequently incubated at 37°C and assessed at 24, 48, and 72 h postinfection. G. mellonella were considered dead when no movement was detected in response to touch or if they showed other signs of death, such as disintegration.

### Statistics.

Statistical analyses were carried out using Prism (version 8; GraphPad Software, Inc.). A paired *t* test was used to test for differences in cell adherence, hemolysis, reproductive fitness, and the proportion of persisters between the two groups (invasive isolates versus colonizing isolates). To compare G. mellonella survival curves, a log-rank test was conducted. Two-tailed *P* values of <0.05 were considered statistically significant.

### Data availability.

Whole-genome sequencing dataset and PacBio SMRT reads of the 22 isolates are available from European Nucleotide Archive project PRJEB47806.
