# Supplementary material for: Limited Adaptation of Staphylococcus aureus during Transition from Colonization to Invasive Infection
Source: Microbiol Spectr. 2023 Jun 21;11(4):e02590-21. doi: 10.1128/spectrum.02590-21 (PMC10433843; doi:10.1128/spectrum.02590-21)
Supplement: Supplemental file 1 — Supplemental material. Download spectrum.02590-21-s0001.pdf, PDF file, 0.4 MB [file spectrum.02590-21-s0001.pdf]

Clinical isolate	P	A	FOX	MOX	NOR	TOB	CN	TE	MI	E	CLI	RA	TEC	STX	DPC	K	CIP	LEV	SPX	PEF	TMP	FA
**CI1814**	**14**	**17**	25	28	25	27	25	30	30	**10**	**26**	33	18	30								
**CI1818**	**17**	**15**	28	26	30	28	25	32	29	**11**	**27**	36	18	28								
**CI2016**	33	34	26	25	29	25	25	31	29	29	28	34	18	32								
**CI2025**	**13**	**14**	25	24	27	25	23	28	27	28	26	30	18	28								
**CI2050**	31	27	27	26	28	26	25	31	27	29	28	34	21	29								
**CI2056**	28	24	23	25	30	24	23	27	27	25	26	32	19	28								
**CI2182**	30	35	28	26	27	27	24	26	25	27	29	35	18	32								
**CI2327**	30	34	24	27	24	27	24	28	26	28	28	32	19	32								
**CI2647**	**10**	**12**	25	24	21	24	22	26	24	25	25	27	18	27								
**CI2670**	**11**	**12**	24	25	24	25	24	27	25	27	27	29	18	26								
**CI3183**	28	28	25	28	27	23	22	28	27	27	28	31	16	26	0.19							
**CI3203**	34	32	24	26	26	24	25	27	26	30	27	32	18	28		19	28	33	24	32		
**CI3984**	28	26	25	24	24	23	22	25	25	28	25	27	17	25								
**CI3990**	30	27	25	26	27	27	24	28	27	31	32	30	19	30		24	25	33	26	32		
**CI4065**	**17**	**17**	28	27	30	24	24	**19**	29	**10**	**37**	35	17	31								
**CI4077**	**12**	**12**	26	27	24	25	24	28	27	32	27	33	19	31		22	25	30	26	32		
**CI4099**	**6**	**6**	**6**	**6**	**6**	27	26	30	28	33	29	34	18	32								
**CI4130**	**6**	**6**	**6**	**6**	**6**	28	27	30	27	30	29	34	19	35		25	**6**	**7**	29	33	**9**	**6**
**CI4090**	**6**	**6**	**13**	**13**	23	**15**	26	**20**	28	**11**	27	33	18	28								
**CI4097**	**6**	**6**	**16**	**15**	24	18	27	**20**	26	**12**	27	35	19	31								
**CI4116**	**13**	**13**	26	27	24	22	23	28	27	31	31	34	19	32								
**CI4152**	**14**	**13**	25	25	26	26	26	29	27	32	29	34	20	33		22	28	33	22	31		

Supplementary Table 1 - Disk diffusion test diameters and antibiotics tested. The *S. aureus* clinical isolates were tested for resistance to the antibiotics listed in the table above. The numbers represent the inhibition zone diameters (in mm) derived from the disk diffusion test. DPC resistance testing was carried out using the E-test (value in ug/ml). Red=resistant, blue=intermediate, black=sensitive. CIP=ciprofloxacin, CLI=clindamycin, P=penicillin, AM=ampicillin, FOX=cefoxitin,

MOX=moxalactym, NOR=norfloxacin, TOB=tobramycin, CN=gentamicin, TE=tetracyclin, MI=minocycline, E=erythromycin, RA=rifampicin, TEC=teicoplanin, STX=Bactrim, DPC=daptomycin, K=kanamycin, LEV=levofloxacin, SPX=sparfloxacin, PEF=perfloxacin, TMP=trimethoprim, FA=fusidic acid.

**Strain**	**ST**	**GenBank accession**	**Reference**
USA400-0051	1	NZ_CP019575	[1]
N315	5	NC_002745.2	[2]
M2024	6	NZ_CP047021	[3]
USA300-FPR3757	8	CP000255	[4]
HO 5096 0412	22	HE681097	[5]
MRSA-M2	30	NZ_AMTC01000001	[6]
MCRF184	45	NZ_CP014791	[7]
AUS0325	88	NZ_LT615218	[8]
MOK063	97	NZ_CP029629	[9]

Supplementary Table 2 – reference strains. Here are listed the publicly available strains used as references for the comparison of the clinical isolates, with their corresponding sequence-types (ST), GenBank accession numbers as well as original publication.

Isolate	Relative to	Position	Eype	Effect	Protein	Gene	Product
CI1814	NZ_CP047021	572249	snp	missense_variant c.128T>C p.Leu43Ser	GQX63_RS02655		amidohydrolase
CI1818	CI1814	515482	del	AT>A			
CI1818	NZ_CP047021	1111102	snp	G>A			
CI1818	NZ_CP047021	1733476	snp	missense_variant c.156A>G p.Ile52Met	GQX63_RS08235		HAD family hydrolase
CI1814	NZ_CP047021	1819665	snp	synonymous_variant c.144T>C p.Phe48Phe	GQX63_RS08625		ImmA/IrrE family metallo-endopeptidase
CI1818	NZ_CP047021	1533158	snp	synonymous_variant c.93G>A p.Thr31Thr	GQX63_RS07225	recN	DNA repair protein RecN
CI1814	NZ_CP047021	375694	snp	synonymous_variant c.324A>G p.Arg108Arg	GQX63_RS01660		bifunctional homocysteine S-methyltransferase/methylenetetrahydrofolate reductase
CI1814	NZ_CP047021	249032	snp	synonymous_variant c.456C>T p.Asp152Asp	GQX63_RS01040		L-lactate dehydrogenase
CI1818	NZ_CP047021	2586598	snp	synonymous_variant c.120G>A p.Ala40Ala	GQX63_RS12895	sdaAA	L-serine ammonia-lyase, iron-sulfur-dependent, subunit alpha
CI1814	NZ_CP047021	2650714	snp	synonymous_variant c.966A>G p.Glu322Glu	GQX63_RS13210		CocE/NonD family hydrolase
CI1814	NZ_CP047021	2532505	snp	missense_variant c.688G>A p.Val230Ile	GQX63_RS12650		membrane protein
CI1818	NZ_CP047021	2474835	snp	stop_gained c.359T>A p.Leu120*	GQX63_RS12355		ABC transporter ATP-binding protein
CI1818	NZ_CP047021	2406983	snp	synonymous_variant c.1113A>G p.Leu371Leu	GQX63_RS12025		L-lactate permease

CI1818	NZ_CP047021	1481903	ins	T>TA			
CI1818	NZ_CP047021	1430782	del	frameshift_variant c.13886delG p.Gly4629fs	GQX63_RS06790	ebh	hyperosmolarity resistance protein Ebh
CI1814	NZ_CP047021	2027060	snp	missense_variant c.1835A>G p.Asp612Gly	GQX63_RS09955		hypothetical protein
CI1818	NZ_CP047021	2080129	snp	missense_variant c.283T>C p.Tyr95His	GQX63_RS10315	agrA	response regulator transcription factor
CI1814	NZ_CP047021	2112775	del	GT>G			
CI1818	NZ_CP047021	2119982	snp	A>G			
CI1818	NZ_CP047021	2172106	snp	missense_variant c.113A>G p.Gln38Arg	GQX63_RS10785		UDP-N-acetylglucosamine 1-carboxyvinyltransferase
CI1814	NZ_CP047021	89373	snp	A>G			
CI1818	NZ_CP047021	1290060	snp	synonymous_variant c.66T>C p.Ile22Ile	GQX63_RS06190		sensor histidine kinase
CI2016	NZ_CP029629	2614266	snp	synonymous_variant c.543C>T p.Gly181Gly	DLJ56_RS13600		hydroxymethylglutaryl-CoA reductase, degradative
CI2025	NZ_CP029629	2153856	snp	synonymous_variant c.15A>G p.Gly5Gly	DLJ56_RS11180	mvk	mevalonate kinase
CI2016	NZ_CP029629	2121258	snp	missense_variant c.566C>T p.Thr189Ile	DLJ56_RS10985		DMT family transporter
CI2025	NZ_CP029629	295778	snp	missense_variant c.1199C>T p.Thr400Ile	DLJ56_RS01315		ribonuclease YeeF family protein
CI2016	NZ_CP029629	159169	snp	synonymous_variant c.594G>A p.Gln198Gln	DLJ56_RS00700		4'-phosphopantetheinyl transferase superfamily protein

CI2025	NZ_CP029629	929336	del	frameshift_variant c.36delT p.Phe12fs	DLJ56_RS05000		hypothetical protein
CI2025	NZ_CP029629	2370593	snp	G>A			
CI2025	NZ_CP029629	2435974	del	disruptive_inframe_deletion c.915_926delTGCAATTGGTGG p.Ala306_Gly309del	DLJ56_RS12685		PTS sucrose transporter subunit IIBC
CI2025	CI2016	25244	snp	A>G			
CI2025	CI2016	17379	snp	C>A			
CI2025	CI2016	11801	snp	A>C			putative lipoprotein
CI2025	CI2016	11811	snp	C>T			putative lipoprotein
CI2025	CI2016	11837	snp	G>A			putative lipoprotein
CI2025	CI2016	11846	snp	T>A			putative lipoprotein
CI2025	CI2016	46766	del	AAT>A			hypothetical protein
CI2025	NZ_CP029629	796734	snp	missense_variant c.167A>G p.Gln56Arg	DLJ56_RS04105	gatA	Asp-tRNA(Asn)/Glu-tRNA(Gln) amidotransferase subunit GatA
CI2016	NZ_CP029629	17890	snp	missense_variant c.85T>C p.Phe29Leu	DLJ56_RS00075	gdpP	cyclic-di-AMP phosphodiesterase GdpP
CI2016	NZ_CP029629	1391365	snp	synonymous_variant c.2166A>G p.Thr722Thr	DLJ56_RS07175	mprF	bifunctional lysylphosphatidylglycerol flippase/synthetase MprF
CI2025	NZ_CP029629	2361765	snp	C>T			
CI2025	NZ_CP029629	2321375	snp	missense_variant c.766T>C p.Ser256Pro	DLJ56_RS12045		efflux RND transporter permease subunit

CI2016	NZ_CP029629	471537	snp	missense_variant c.128G>C p.Arg43Pro	DLJ56_RS02390		hypothetical protein
CI2056	NZ_CP014791	985658	snp	missense_variant c.406G>T p.Gly136Cys	CKU_RS04795		phosphoribosylformylglycinamidine cyclo-ligase
CI2056	NZ_CP014791	718203	del	frameshift_variant c.105_117delCTTACCAACTGGT p.Leu36fs	CKU_RS03475	recQ	DNA helicase RecQ
CI2050	NZ_CP014791	1482158	snp	synonymous_variant c.378A>G p.Gly126Gly	CKU_RS07150	proC	pyrroline-5-carboxylate reductase
CI2050	NZ_CP014791	1158323	snp	synonymous_variant c.90T>A p.Arg30Arg	CKU_RS05675	rimM	ribosome maturation factor RimM
CI2056	NZ_CP014791	1173973	snp	missense_variant c.889G>A p.Gly297Ser	CKU_RS05740		methylenetetrahydrofolate--tRNA-(uracil(54)-C(5))-methyltransferase (FADH (2)-oxidizing) TrmFO
CI2056	NZ_CP014791	1215125	snp	synonymous_variant c.894T>G p.Gly298Gly	CKU_RS05920	recA	recombinase RecA
CI2050	NZ_CP014791	372725	snp	A>T			
CI2182	CP000255	662829	snp	synonymous_variant c.69A>G p.Ala23Ala	SAUSA300_0589		aldo/keto reductase family protein
CI2182	CP000255	681310	snp	synonymous_variant c.87C>T p.Phe29Phe	SAUSA300_0608		conserved hypothetical protein
CI2182	CP000255	714036	snp	T>C			
CI2182	CP000255	968216	snp	synonymous_variant c.34C>A p.Arg12Arg	SAUSA300_0881		putative membrane protein

CI2327	CP000255	1024038	snp	missense_variant c.572C>T p.Ala191Val	SAUSA300_0934		membrane protein
CI2182	CP000255	1263673	snp	missense_variant c.200C>T p.Ser67Phe	SAUSA300_1152	rrf	ribosome recycling factor
CI2182	CP000255	1789880	snp	missense_variant c.731G>A p.Arg244His	SAUSA300_1635	mutM	formamidopyrimidine-DNA glycosylase
CI2182	CP000255	1683455	snp	synonymous_variant c.219A>G p.Thr73Thr	SAUSA300_1533		conserved hypothetical protein
CI2327	CP000255	1681966	del	AT>A			
CI2327	CP000255	1651324	snp	missense_variant c.271A>G p.Ile91Val	SAUSA300_1497		glycine dehydrogenase, subunit 1 (glycine cleavage system P protein)
CI2327	CP000255	1509350	snp	missense_variant c.11A>C p.Tyr4Ser	SAUSA300_1344		putative DNA replication protein DnaD
CI2327	CP000255	2766070	snp	G>T			
CI2182	CP000255	282563	snp	A>G			
CI2327	CP000255	194570	snp	missense_variant c.500C>T p.Ala167Val	SAUSA300_0171		cation efflux family protein
CI2327	CP000255	2034306	snp	G>A			
CI2327	CI2182	63478	del	TA>T			
CI2327	CI2182	99176	snp	T>C			
CI2327	CP000255	436071	snp	A>G			
CI2182	CP000255	402701	snp	missense_variant c.203A>G p.Glu68Gly	SAUSA300_0352		ABC transporter, ATP-binding protein
CI2327	CP000255	394277	snp	missense_variant c.461G>T p.Ser154Ile	SAUSA300_0341		putative membrane protein

CI2327	CI2182	38140	snp	A>G			tRNA-Val(tac)
CI2327	CP000255	1880776	ins	frameshift_variant c.3692dupA p.Asn1231fs	SAUSA300_1702		cell wall surface anchor family protein
CI2327	CP000255	2191419	snp	synonymous_variant c.852G>A p.Ser284Ser	SAUSA300_2030		putative membrane protein
CI2182	CP000255	2195295	snp	missense_variant c.209G>T p.Gly70Val	SAUSA300_2033	kdpB	K+-transporting ATPase, B subunit
CI2182	CP000255	2210698	snp	G>A			
CI2327	CP000255	599382	snp	C>G			
CI2670	NZ_LT615218	397029	snp	synonymous_variant c.252A>G p.Gly84Gly	BQ3358_RS01850		superantigen-like protein SSL10
CI2647	NZ_LT615218	2014882	snp	synonymous_variant c.171T>A p.Gly57Gly	BQ3358_RS10195		DUF1381 domain-containing protein
CI2647	NZ_LT615218	411787	snp	C>G			
CI3203	NZ_CP019574	475022	snp	synonymous_variant c.567G>A p.Glu189Glu	SAU400_RS02365		LysR family transcriptional regulator

Supplementary Table 3 - Variants between pairs of closely related isolates. Out of the 10 pairs, 4 were identical (CI3990-CI3984, CI4097-CI4090, CI4130-CI4099, CI4152-CI4116). The other six pairs harbor between 1 and 26 variants. Variants found only between the closely related isolates but not when comparing them to the reference strain are reported, without details on their effect. Non-synonymous changes are highlighted in grey. Variants for each pair are annotated relative to the corresponding closest reference strain (see Tab.S2).

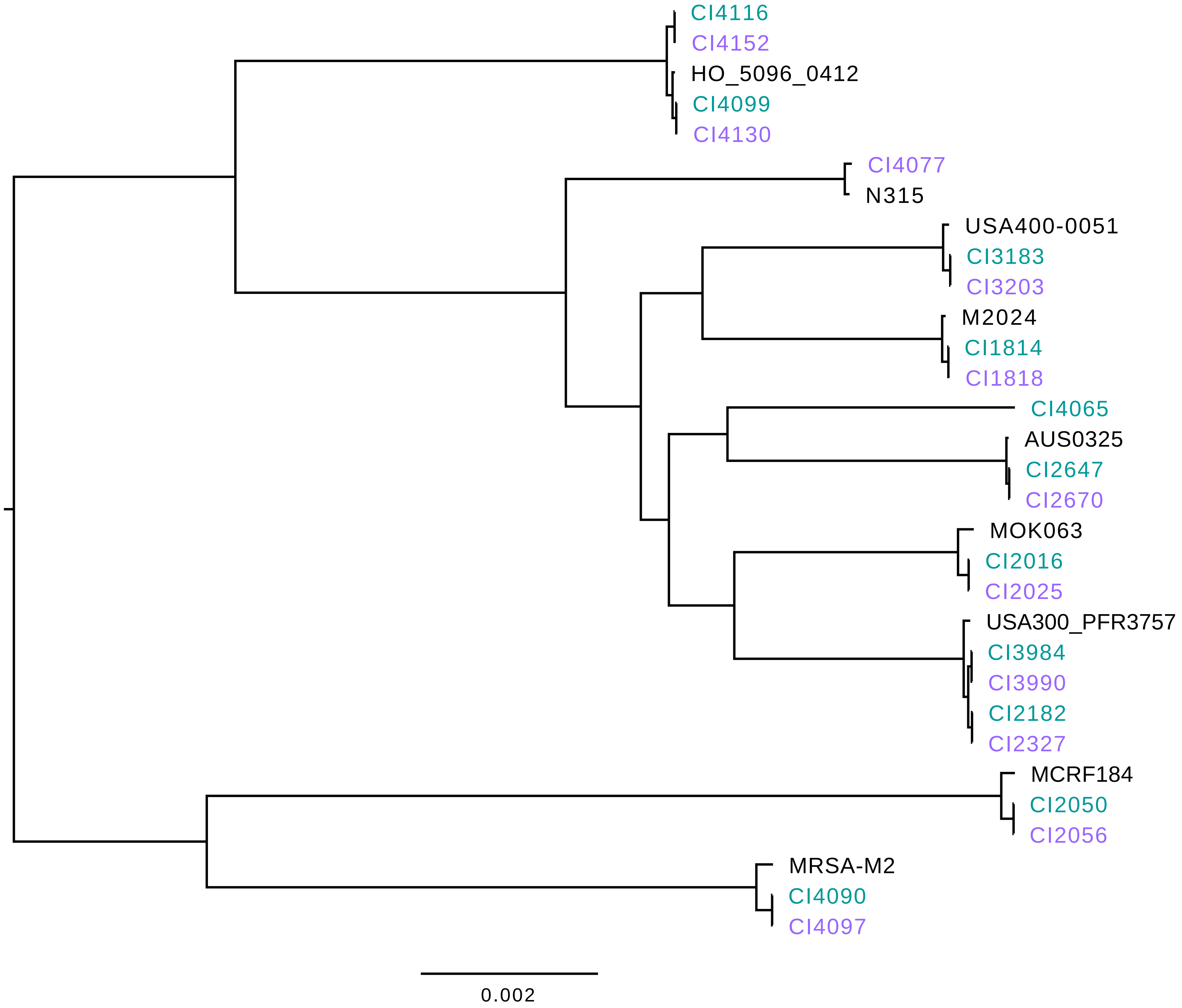
CI4116
CI4152
HO_5096_0412
CI4099
CI4130
CI4077
N315
USA400-0051
CI3183
CI3203
M2024
CI1814
CI1818
CI4065
AUS0325
CI2647
CI2670
MOK063
CI2016
CI2025
USA300_PFR3757
CI3984
CI3990
CI2182
CI2327
MCRF184
CI2050
CI2056
MRSA-M2
CI4090
CI4097
0.002

Supplementary Figure 1 - Phylogenetic tree. Midpoint rooted maximum likelihood phylogenetic tree based on the alignment of the clinical isolates and reference strains core genes (totaling 1’797’434 sites). The scale bar corresponds to 0.002 substitutions/site, e.g. approximatively 3’600 substitutions. Invasive isolates are labeled in turquoise and colonizing isolates in purple. Variants found only between the closely related isolates but not when comparing them to the reference strain are reported.

Persistence log phase

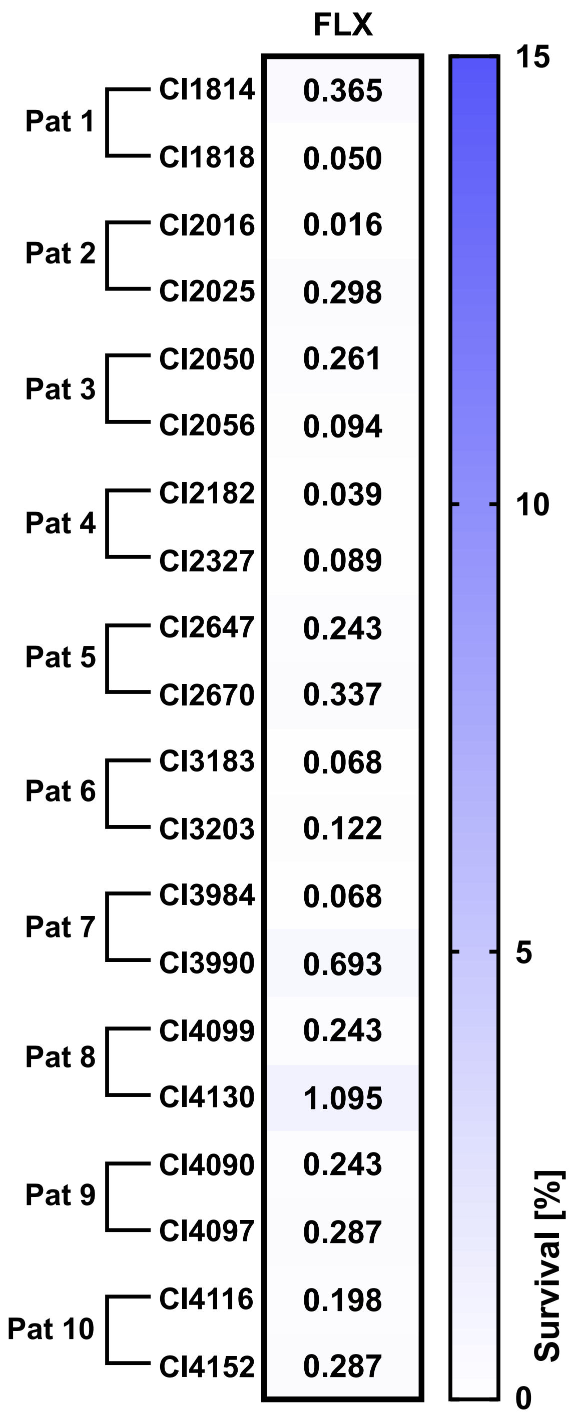

Supplementary Figure 2 – Persisters levels in logarithmic phase growth cultures. Persisters formation was assessed on *S. aureus* strains after growth in TSB to logarithmic phase followed by incubation for 24h in pH 7.4 medium in the presence or absence of 40 times the minimal inhibitory concentration (MIC) of flucloxacillin (FLX). After 24h the antibiotics were washed out and the surviving bacteria were plated on TSB plates and counted. The figure shows the surviving bacteria as a percentage of the inoculum. At least 3 biological replicates were carried out per strain.

References

1. Côrtes, M.F., et al., *Complete genome sequence of community-associated methicillin-resistant Staphylococcus aureus (strain USA400-0051), a prototype of the USA400 clone.* Mem Inst Oswaldo Cruz, 2017. **112**(11): p. 790-792.
2. Kuroda, M., et al., *Whole genome sequencing of meticillin-resistant Staphylococcus aureus.* Lancet, 2001. **357**(9264): p. 1225-40.
3. Bartels, M.D., et al., *Repeated introduction and spread of the MRSA clone t304/ST6 in northern Europe.* Clin Microbiol Infect, 2020.
4. Diep, B.A., et al., *Complete genome sequence of USA300, an epidemic clone of community-acquired meticillin-resistant Staphylococcus aureus.* Lancet, 2006. **367**(9512): p. 731-9.
5. Holden, M.T., et al., *A genomic portrait of the emergence, evolution, and global spread of a methicillin-resistant Staphylococcus aureus pandemic.* Genome Res, 2013. **23**(4): p. 653-64.
6. Harro, J.M., et al., *Draft Genome Sequence of the Methicillin-Resistant Staphylococcus aureus Isolate MRSA-M2.* Genome Announc, 2013. **1**(1).
7. Aswani, V., B. Mau, and S.K. Shukla, *Complete Genome Sequence of Staphylococcus aureus MCRF184, a Necrotizing Fasciitis-Causing Methicillin-Sensitive Sequence Type 45 Staphylococcus Strain.* Genome Announc, 2016. **4**(3).
8. Kpeli, G., et al., *Genomic analysis of ST88 community-acquired methicillin resistant Staphylococcus aureus in Ghana.* PeerJ, 2017. **5**: p. e3047.

9. Cormican, P. and O.M. Keane, *Complete Genome Sequences of Sequence Type 71 (ST71) and ST97 Staphylococcus aureus Isolates from Bovine Milk.* Microbiol Resour Announc, 2018. **7**(5).
